# Anomalous Water Flow in Sub‐Nanometer Carbon Nanoconfinement

**DOI:** 10.1002/smll.202508637

**Published:** 2025-10-16

**Authors:** Md Masuduzzaman, Chirodeep Bakli, Murat Barisik, BoHung Kim

**Affiliations:** ^1^ Robert F. Smith School of Chemical and Biomolecular Engineering Cornell University Ithaca NY 14853 USA; ^2^ School of Mechanical Engineering University of Ulsan Daehak‐ro 93, Namgu Ulsan 680–749 South Korea; ^3^ School of Energy Science and Engineering Indian Institute of Technology Kharagpur Kharagpur West Bengal 721302 India; ^4^ Mechanical Engineering Department University of Tennessee at Chattanooga Chattanooga TN 37403 USA

**Keywords:** hydrogen bond dynamics, molecular dynamics simulations, Pauli exclusion effects, Pseudo‐continuum behavior, single‐file transport

## Abstract

Sub‐nanometer confinement fundamentally transforms fluid transport phenomena by introducing pronounced molecular‐scale interactions that challenge the validity of classical hydrodynamic models. Utilizing advanced molecular dynamics simulations, this study systematically analyzes the transport properties of Lennard‐Jones fluids and polar water within carbon nanotubes and graphene‐based nanochannels. In these ultra‐confined regimes, where molecular discreteness dominates and continuum constructs such as viscosity and velocity profiles become ambiguous, this study observes that water exhibits flow velocities up to threefold higher than Lennard‐Jones fluids, quantitatively consistent with recent experimental and computational observations in sub‐nanometer confinement. This anomalous transport originates from the disruption of hydrogen‐bond networks, intensified interfacial Pauli exclusion forces, and a reduction in effective viscosity. Curvilinear geometries, typified by carbon nanotubes, induce enhanced molecular ordering and facilitate accelerated mass flux, while planar nanochannels impose steric constraints that suppress transport efficiency. The intrinsically lower molecular mass of water further augments its dynamic response under nanoscale confinement. Notably, despite the breakdown of classical assumptions, continuum‐like velocity profiles emerge in specific regimes, signifying the onset of pseudo‐continuum behavior at the molecular scale. These results elucidate the fundamental physical origins of confinement‐induced transport anomalies and establish a predictive theoretical framework for extending continuum fluid concepts into the atomistic regime.

## Graphical abstract

Ultra‐narrow carbon nanoconfinement enables single‐file transport and interface‐governed flow, driving superfast water mobility. Disrupted hydrogen bonding and intensified molecular ordering underpin anomalous dynamics, bridging atomistic insight with pseudo‐continuum behavior. These results illuminate how sub‐nanometer channels fundamentally redefine fluid transport, inspiring next‐generation nanofluidic devices.

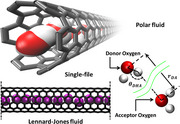

## 1 Introduction

Fluid transport through sub‐nanometer (sub‐nm) confinements exhibits highly anomalous behavior, significantly challenging classical hydrodynamic predictions due to the dominance of molecular interactions and interfacial effects.^[^ ^1^, ^2^ ^]^ The extreme surface‐to‐volume ratio at this scale amplifies solid–fluid interactions, profoundly altering the thermodynamic and structural properties of confined fluids.^[^ ^1^, ^3^ ^]^ These effects include surface slippage,^[^ ^4^ ^]^ molecular confinement‐induced ordering,^[^ ^2^ ^]^ curvature‐driven phenomena,^[^ ^5^ ^]^ and the formation of hydration layers at interfaces,^[^ ^6^ ^]^ collectively leading to fluid behaviors not observed in the bulk phase. These transport characteristics enable applications in water desalination, ionic diodes, semiconductors, water purification, drug delivery, and lab‐on‐a‐chip systems.^[^ ^7^ ^]^ Furthermore, discrepancies between continuum‐based predictions and molecular dynamics (MD) simulations underscore the need to refine theoretical models capable of accurately capturing molecular‐scale phenomena, including van der Waals and Coulombic interactions, hydration layers, and molecular ordering at interfaces. Under sub‐nm confinement, fluids exhibit pseudo‐continuum behavior, where transport partially retains continuum‐like characteristics despite molecular effects. These deviations highlight the fundamental limitations of classical models and the need to investigate the molecular mechanisms governing fluid transport at the sub‐nm scale.

To address these fundamental gaps, extensive experimental, MD, and quantum mechanical studies have been conducted to elucidate fluid behavior at sub‐nm scales. Experiments consistently reveal anomalously fast water transport through sub‐2‐nm carbon nanotube (CNT) membranes, where flow rates exceed continuum predictions by several orders of magnitude, indicating the breakdown of classical hydrodynamics at this scale.^[^ ^8^, ^9^, ^10^ ^]^ Qin et al. demonstrated enhanced water permeability in sub‐2‐nm CNTs, while similar flow enhancements were observed in macroscopic membranes containing sub‐1‐nm CNT pores.^[^ ^8^, ^9^ ^]^ However, contrasting results by Karan et al. reported Hagen–Poiseuille‐consistent flow in ≈1 nm diamond‐like carbon pores, highlighting the intricate dependence of transport behavior on pore surface chemistry and fluid structure.^[^ ^11^ ^]^ MD simulations revealed that water confined in sub‐nm CNTs, such as (6,6), forms single‐file chains with dipole alignment,^[^ ^12^ ^]^ while experimental measurements showed transport rates nearly an order of magnitude higher than those in aquaporins.^[^ ^13^ ^]^ These chains exhibit unique thermodynamic and dynamic properties, including quasi‐phase transitions and dual diffusion states.^[^ ^14^, ^15^ ^]^ Complementary quantum mechanical studies predict confinement‐induced quantum phase transitions, suggesting that electronic and quantum effects critically influence flow behavior at this scale.^[^ ^16^ ^]^ Despite these insights, both experimental and computational findings report significantly reduced viscosity and enhanced flow velocity under sub‐nm confinement, phenomena that continuum models fail to capture.^[^ ^17^ ^]^ Existing theoretical frameworks inadequately account for steric hindrance, dielectric screening, and energy barriers at interfaces—effects that dominate at molecular scales. This persistent modeling gap underscores the need for integrated multiscale approaches to resolve the mechanisms governing fast transport and low‐friction flow in sub‐nm confinements, beyond the scope of classical hydrodynamics.^[^ ^18^ ^]^

Understanding the molecular mechanisms governing fluid transport in sub‐nm confinement requires detailed resolution of interfacial phenomena, including density layering, molecular ordering, slip behavior, and local viscosity variations—features that are inaccessible to continuum approaches. While experimental techniques provide critical flow measurements, challenges in directly probing molecular‐scale interactions and local transport properties within angstrom‐scale channels limit their ability to resolve these interfacial mechanisms.^[^ ^10^ ^]^ Quantum mechanical methods, though capable of capturing electronic effects, are computationally prohibitive for large fluid systems over meaningful timescales.^[^ ^19^ ^]^ In contrast, MD simulations offer a unique balance of atomistic detail and computational tractability, enabling explicit modeling of particle‐particle interactions, molecular structuring, and dynamic flow behaviors at the sub‐nm scale.^[^ ^20^ ^]^ Earlier computational methods, such as grand canonical Monte Carlo simulations,^[^ ^21^ ^]^ classical density functional theory,^[^ ^22^ ^]^ and continuum hydrodynamics^[^ ^5^ ^]^ provided insights into adsorption, density profiles, and macroscopic transport properties, but lack the resolution needed to capture dynamic interfacial phenomena under extreme confinement. MD simulations overcome these limitations, resolving time‐dependent molecular rearrangements, van der Waals interactions, and confinement‐induced structuring essential for accurately characterizing flow behavior in carbon nanotube and graphene nanochannels. This study employs MD simulations to quantify these effects under pressure‐driven conditions, bridging the gap between molecular‐scale mechanisms and continuum breakdown, and providing insights necessary for developing predictive models of fluid transport in sub‐nm systems.

Water transport through sub‐nm confinements is fundamentally governed by molecular‐scale interactions that drive deviations from classical hydrodynamics. In CNTs narrower than 1 nm, spatial confinement enforces single‐file water alignment, enhancing diffusion due to reduced rotational freedom and steric constraints.^[^ ^23^, ^24^ ^]^ This contrasts with rectangular graphene nanochannels, where the planar geometry disrupts the hydrogen‐bond (H‐bond) network and increases flow resistance.^[^ ^25^ ^]^ Under confinement, water molecules reorient their dipoles along the flow direction, saturating at high velocities and modulating the energy landscape of transport.^[^ ^26^ ^]^ Notably, in CNTs of 3.0–4.5 nm diameter, diffusion becomes non‐Arrhenius, reflecting a competition between thermal agitation and confinement‐induced ordering.^[^ ^27^ ^]^ These effects are accompanied by a substantial viscosity reduction inside narrow channels, as limited H‐bond formation and suppressed molecular degrees of freedom lower internal resistance.^[^ ^28^ ^]^ Experimental studies further reveal that water flow can be amplified by over two orders of magnitude in membranes with sub‐1‐nm CNT pores, emphasizing the inadequacy of continuum assumptions at this scale.^[^ ^9^ ^]^ However, hydrophilic surfaces intensify interfacial H‐bonding, increasing local viscosity and creating heterogeneous flow profiles near the walls.^[^ ^29^, ^30^ ^]^ Concurrently, slip length rises sharply as channel dimensions shrink and surfaces smoothen, reflecting reduced momentum transfer and friction at the solid‐fluid interface.^[^ ^31^ ^]^ Under extreme confinement, water molecules exhibit solid‐like ordering and restricted translational motion, reorganizing H‐bonds and increasing interfacial friction. This structured layering subjects molecules to simultaneous van der Waals (vdW) attractions and Coulombic forces, producing viscosity gradients and altering flow stability. Such molecular interactions dominate transport behavior, causing the breakdown of continuum models like Navier‐Stokes, especially in charged systems where electrostatic interactions further complicate flow dynamics.^[^ ^19^ ^]^ Additionally, the cylindrical symmetry and atomically smooth surfaces of CNTs minimize frictional dissipation, enabling superlubricated transport, while planar graphene nanochannels impose greater resistance due to enhanced wall‐molecule collisions.^[^ ^8^, ^24^ ^]^ Despite these findings, a comprehensive framework capturing the coupled effects of molecular distribution, viscosity variation, H‐bond dynamics, and long‐range forces remains lacking. Addressing this gap, the present study employs molecular dynamics simulations to quantify local viscosity fluctuations, hydrogen‐bond reorganization, and the interplay of van der Waals and Coulombic forces, offering detailed insights into pseudo‐continuum behavior and continuum breakdown in pressure‐driven water transport through sub‐nanometer CNTs and graphene nanochannels.

Beyond simple molecular fluids, ionic solutions and responsive molecular fluids exhibit unique transport behaviors at sub‐nanometer scales. Y. Hou and X. Hou,^[^ ^32^ ^]^ together with Elverfeldt et al.,^[^ ^33^ ^]^ stated that ions under confinement experience altered hydration shells, charge screening, selective transport, and size exclusion effects, which are critical for applications such as desalination, energy harvesting, and biosensing. Lv, Shurui, et al.^[^ ^34^ ^]^ and Li, Bofeng, et al.^[^ ^35^ ^]^ reported that responsive fluids demonstrate adaptive molecular rearrangement and tunable transport properties in response to external stimuli, enabling smart control over nanoscale fluidics. These complex transport phenomena underscore the significance of understanding confined water transport since water's hydrogen bonding and polarity fundamentally influence ion and responsive molecule behavior in nanoconfined environments. Consequently, elucidating water transport mechanisms lays a critical foundation for advancing nanofluidic applications involving diverse fluid types.

In this study, molecular dynamics simulations are employed to systematically elucidate the structural and transport properties of Lennard‐Jones (LJ) fluids and water confined within sub‐nm CNT and graphene nanochannels, where classical continuum theory fundamentally fails to capture the governing physics. The analysis quantifies molecular density distributions, diffusion coefficients, velocity profiles, and hydrogen‐bond network perturbations induced by extreme spatial confinement. Comparative assessment of cylindrical and planar topologies reveals how confinement geometry and interfacial interactions orchestrate molecular organization and engender pronounce deviations from bulk transport behavior. Cylindrical confinement promotes axially aligned molecular layering and uniform velocity fields, whereas planar graphene channels induce spatial heterogeneity and anisotropic transport due to intensified wall‐fluid coupling. The results delineate the emergence of a previously uncharacterized pseudo‐continuum regime; wherein discrete molecular interactions govern flow dynamics while mesoscopic features reminiscent of continuum systems persist. By explicitly incorporating van der Waals and Coulombic interactions, this work establishes a predictive molecular‐level framework for interpreting transport phenomena in ultra‐confined environments. These findings resolve a fundamental gap in theoretical understanding by providing mechanistic insight essential for advancing models of fluid behavior at the molecular scale and for guiding the rational design of next generation nanofluidic and membrane technologies.

## 2 Theory and Computational Simulation

### 2.1 Theoretical Background

Pressure‐driven fluid transport through cylindrical nanochannels is commonly described by the Hagen–Poiseuille (HP) equation, derived from the continuum Navier–Stokes (NS) equations.^[^ ^36^, ^37^ ^]^ The classical HP model applies under the following assumptions: i) the fluid is incompressible, Newtonian, and in a steady state $\left(\nabla .\rho \;\vec{V}=0,\;\mathrm{at}\;\mathrm{constant}\;\mathrm{density}\;\mathrm{and}\;\mathrm{uniform},\;\nabla .\;\vec{V}=0\right)$ with zero net acceleration; ii) the flow is unidirectional, laminar, and fully developed along the axial direction; iii) the pipe length is substantially larger than its diameter, minimizing entrance effects; and iv) viscous forces dominate over inertial effects. These assumptions are often violated in nanoconfined systems, where the length scales approach molecular dimensions and interfacial phenomena dominate. In particular, the no‐slip boundary condition at the solid–fluid interface frequently breaks down, and confined fluids may exhibit viscosities distinct from bulk values due to molecular layering, structural ordering, and strong fluid–solid interactions.^[^ ^38^ ^]^

Under these conditions, the HP velocity profile is expressed as:

(1) $$V_{z,r\left(HP\right)}=\frac{R^{2}}{4\mu }\left(1-\frac{r^{2}}{R^{2}}\right)\frac{\delta P}{\delta Z}$$

where μ is the viscosity, *R* is the tube radius, and δ*P*/δ*Z* is the applied pressure gradient.

At the nanoscale, however, both the no‐slip boundary condition and the assumption of bulk viscosity require modification. MD simulations and experiments consistently show that fluid molecules exhibit a finite slip velocity at the wall (*r*  =  *R*),^[^ ^39^ ^]^ while the effective viscosity may deviate from the bulk value. The HP model can therefore be extended to include a wall slip velocity *V_s_*;

(2) $$V_{z,r\;\left(MHP\right)}=\frac{R^{2}}{4\mu }\left(1-\frac{r^{2}}{R^{2}}\right)\frac{\delta P}{\delta Z}+V_{s}$$

This modified HP (MHP) form incorporates essential nanoscale corrections, including interfacial slip. The slip length *L_s_* can then be explicitly quantified from simulation data as:

(3) $$L_{s}=\frac{V_{s}}{{\left.\frac{\delta V}{\delta z}\right|}_{wall}}$$

which relates the observed slip velocity to the velocity gradient at the wall. In practice, velocity profiles obtained from MD simulations are fitted to a generalized parabolic form,

(4) $$V\left(r\right)=ar^{2}+c$$

where *a* and *c* are fitting coefficients. By comparing this expression with the continuum HP form, the coefficient *a* is directly related to the effective or pseudo viscosity μ_*pseudo*_ of the confined fluid, defined as:

(5) $$a=\frac{-1}{4\mu_{pseudo}}\frac{\delta P}{\delta Z}$$

Rearranging gives the pseudo viscosity as:

(6) $$\mu_{pseudo}=\frac{-1}{4a}\frac{\delta P}{\delta Z}$$

Together, Equations (3)‐(6) enable the extraction of both slip length and pseudo viscosity from molecular simulations, providing an effective bridge between continuum theory and nanoscale transport behavior. For the (6,6) CNT studied here (diameter ≈0.8 nm), we obtain slip lengths of ≈3.5 −4.5 *nm*, consistent with previous reports for water flow through carbon‐based nanoconduits.^[^ ^40^ ^]^

Furthermore, the validity of the quantified slip length is supported qualitatively by observations related to depletion length near the channel wall reported in the literature. Studies have shown that water molecules form distinct high‐density layers near hydrophilic surfaces, while depletion zones emerge near hydrophobic interfaces depending on surface energy and wettability.^[^ ^41^, ^42^, ^43^, ^44^ ^]^ In regions where the depletion length is small, indicating denser molecular packing, the slip velocity is suppressed due to stronger fluid–solid interactions and increased structural ordering. Conversely, larger depletion zones correspond to weaker interfacial forces, allowing for higher slip velocities as the fluid molecules experience less hindrance at the wall. These depletion phenomena highlight how nanoscale interfacial structuring governs the extent of slip, providing a microscopic rationale for the deviations from classical no‐slip boundary conditions. As a result, the observed relationship between depletion length and slip velocity offers qualitative support for the sub‐continuum slip description, where the continuum assumptions are modified to account for molecular layering, density fluctuations, and interfacial energetics at near‐atomic length scales. Such structuration effects have been demonstrated to significantly influence transport behavior, thereby validating the use of slip length as an effective, though not literal, parameter in describing fluid flow in highly confined geometries.^[^ ^41^, ^42^, ^43^, ^44^ ^]^ In the regime of sub‐nanometer CNTs and graphene slits, the continuum‐with‐slip description becomes increasingly approximate and departs from the actual transport behavior captured by MD simulations. At these scales, velocity profiles deviate from ideal parabolas, and transport is strongly influenced by molecular discreteness, single‐file water structures, and hydrogen‐bond networks. Thus, while *L_s_* and μ_*pseudo*_ remain useful coarse‐grained descriptors for comparing MD results with continuum predictions, they should not be interpreted as literal continuum parameters. Instead, they provide effective measures that highlight the extent to which confinement and interfacial interactions reshape fluid transport beyond classical hydrodynamic expectations.

### 2.2 Computational Details

**Figure 1:**
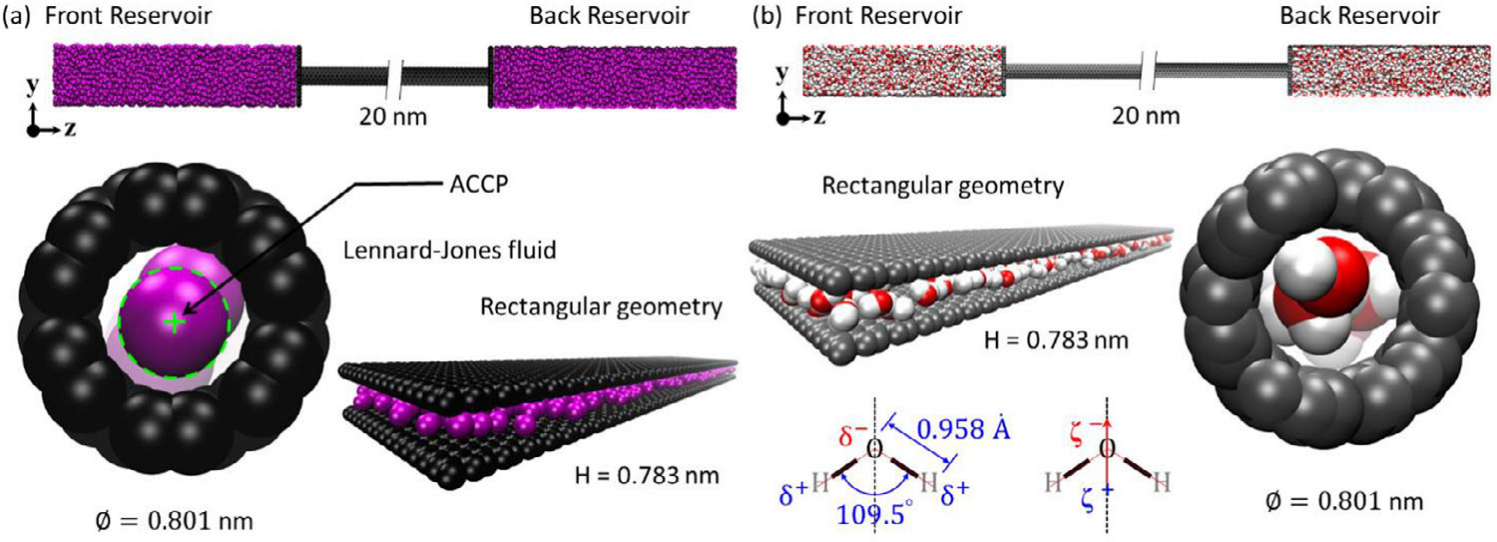
Schematic of sub‐nanometer confinement geometries. Molecular models of a) carbon nanotube (CNT) and rectangular graphene nanochannel filled with Lennard‐Jones (LJ) fluid, and b) CNT and nanochannel filled with water. These configurations were used to investigate fluid transport and molecular structuring under extreme confinement in molecular dynamics simulations.

Molecular dynamics simulations examine the transport behavior of the LJ fluid and polar water under extreme nanoconfinement within CNTs and rectangular graphene nanochannels. As shown in **Figure** **1**, both fluids flow from the front reservoir (FR) to the back reservoir (BR) through channels constructed from armchair (6,6) CNTs or graphene sheets, each 20 nm in length. The CNTs have a diameter of ≈0.8 nm and exhibit metallic character with a zero electronic band gap at the Fermi level, where delocalized electrons influence interfacial energy landscapes relevant to molecular transport.^[^ ^45^ ^]^ The graphene nanochannel provides comparable geometric confinement. Reservoirs extend ≈20 nm along the z‐axis and are bounded by graphene membranes (GMs), while periodic boundary conditions apply in the x‐ and y‐directions with a finite z‐axis to preserve confinement. Bulk densities of 0.8 for the LJ fluid and 1.0 g cm^−^^3^ for water are maintained in the reservoirs, containing 3930–7860 LJ atoms or 6748–7667 water molecules depending on the system, while density variations naturally arise inside the channels due to molecular‐scale effects. Consistent simulation procedures, boundary conditions, and integration schemes are applied throughout, with extended equilibration periods required for water to account for strong intermolecular forces and hydrogen bonding networks. The simulations achieve thermodynamic equilibrium and ergodicity, enabling statistically reliable sampling of phase space. Interactions are modeled using empirical LJ and Coulombic potentials to capture both bulk and confined fluid behavior. Pressure‐driven flow is generated using specular reflection walls (SRWs) positioned at the reservoir ends along the xy‐plane,^[^ ^46^ ^]^ providing a computationally efficient method to apply momentum without introducing artificial pressure gradients. Initially, SRWs are placed 14 nm from the GMs along the z‐axis, and flow is driven by advancing the front SRW while retracting the back SRW, maintaining consistent system conditions across simulations.

In addition to flow control, molecular interactions are governed by interaction potentials designed to accurately represent fluid‐fluid and fluid‐wall interactions. The simple fluid is modeled using a truncated LJ (12‐6) potential, describing van der Waals forces between fluid atoms.

(7) $$V_{truncated}\;\left(r_{ij}\right)=4\varepsilon \left[\left\{{\left(\frac{\sigma }{r_{ij}}\right)}^{12}-{\left(\frac{\sigma }{r_{ij}}\right)}^{6}\right\}-\left\{{\left(\frac{\sigma }{r_{c}}\right)}^{12}-{\left(\frac{\sigma }{r_{c}}\right)}^{6}\right\}\right]$$

where ε represents the depth of the potential well (eV), *r_ij_* is the intermolecular separation, σ is the molecular diameter, and *r_c_* is the cutoff radius, set to 1.0 nm (≈3σ). This formulation ensures that short‐range repulsive and attractive interactions are well captured while truncating longer‐range forces beyond the physical influence of the molecules. The interactions between carbon atoms in the CNTs are modeled using the adaptive intermolecular reactive empirical bond order (AIREBO) potential.^[^ ^47^ ^]^ Fluid‐wall interactions between the simple fluid or water and the CNT or graphene membranes are computed using the Lorentz‐Berthelot mixing rules to determine cross‐interaction parameters.^[^ ^48^ ^]^ All interaction parameters are taken from ref.[49, 50, 51, 52]

For the polarized water simulations, intermolecular interactions included both LJ forces and long‐range Coulombic interactions.^[^ ^53^ ^]^ The SPC/E model is employed due to its established accuracy in reproducing the thermodynamic and transport properties of water. While the LJ potential was applied to the oxygen atoms, Coulombic interactions are calculated using the standard electrostatic potential:

(8) $$\;V_{Coulomb}\;\left(r_{ij}\right)=\frac{1}{4\pi \varepsilon_{0}}\;\frac{q_{i}q_{j}}{r_{ij}}$$

where *q_i_* and *q_j_* are the atomic charges, and ε_0_ is the permittivity of free space. The assigned atomic charges were *q_O_* =   − 0.8476*e* for oxygen and *q_H_* =  0.4238*e* for hydrogen. Due to the significantly lower mass and size of hydrogen atoms, their LJ contribution is neglected. The SHAKE algorithm^[^ ^54^ ^]^ is employed to constrain the H‐O‐H angle to 109.47° and the O‐H bond length to 0.1 nm, preserving the structural integrity of water molecules throughout the simulation. To minimize dipole artifacts arising from periodic boundaries, long‐range electrostatic interactions are evaluated using the Particle‐Particle Particle‐Mesh (PPPM) method.^[^ ^55^ ^]^

MD simulations are conducted using the LAMMPS package,^[^ ^56^ ^]^ integrating Newton's equations of motion with the velocity Verlet algorithm^[^ ^55^ ^]^ and a time step of 1.0 fs. All velocity and viscosity values reported in this study are averaged over three independent simulations. Error bars in the figures represent the standard deviation from these replicates, ensuring statistical reliability of the results. Each simulation ran for 100 ns, with the initial 20 ns allocated for system equilibration under the NVT ensemble (constant number of particles, volume, and temperature) at 100 K for the LJ fluid and 300 K for the polarized water system. These temperatures were chosen based on established phase diagrams that confirm the fluid‐phase stability of LJ fluids near 100 K^[^ ^57^ ^]^ and SPC/E water at 300 K,^[^ ^58^ ^]^ ensuring physically meaningful confinement effects in our simulations. The simulation time of 100 ns is chosen to ensure adequate sampling of molecular transport properties and structural dynamics within nanoconfinement. Although extended simulations exceeding 100 ns are also performed, a duration of 100 ns is sufficient to yield statistically stable results while remaining computationally tractable. This duration allows the system to reach steady‐state flow conditions and equilibrium molecular arrangements, as confirmed by monitoring time convergence of velocity profiles, hydrogen bond statistics, and other relevant observables. Prior molecular dynamics studies of nanoconfined fluids have demonstrated that tens to hundreds of nanoseconds are sufficient to capture these phenomena reliably. The system size is selected to represent realistic confinement geometries while balancing computational feasibility; it is large enough to minimize finite size and boundary effects, ensuring well‐defined molecular layering and interaction characteristics typical of sub‐nm channels. Convergence tests performed on key parameters further validated the adequacy of the chosen system size and simulation duration, providing confidence in the robustness and accuracy of the reported results. Initial velocities are assigned according to the Maxwell‐Boltzmann distribution, and temperature control is maintained using the Nosé‐Hoover thermostat. The final 80 ns is performed in the NVE ensemble while applying pressure along the positive z‐direction to drive the flow. Data collection commences after a 2 ns stabilization period and is averaged over 1 ns interval to improve statistical reliability.

The local stress tensor is evaluated using the Irving‐Kirkwood (IK) formalism,^[^ ^59^, ^60^ ^]^ which incorporates both kinetic and virial contributions. The stress tensor is expressed as:

(9) $$S_{\alpha \beta }=\frac{1}{V}\langle \;\sum_{i}m^{i}\left(v_{\alpha }^{i}-u_{\alpha }\right)\left(v_{\beta }^{i}-u_{\beta }\right)+\frac{1}{2}\sum_{i,j}^{N}\left(r_{\alpha }^{i}-r_{\beta }^{i}\right)f_{\beta }^{i,j}\rangle$$

The first term represents the kinetic contribution, where, *m* represents the mass of atom *i*, *v* stands for the velocity of atom *i*, and *u* corresponds to the streaming velocity in the α and β directions within the Cartesian coordinate system. As for the virial part, $\left(r_{\alpha }^{i}-r_{\beta }^{i}\right)$ signifies the relative distance vector between atom *i* and atom *j*, while $f_{\beta }^{i,j}$ characterizes the intermolecular force exerted on atom *i* by atom *j*.

## 3 Results and Discussion

Figure 1 illustrates the computational models developed to investigate fluid transport through sub‐nm confinements, including a rectangular graphene nanochannel and a (6,6) CNT, each providing an effective confinement width of ≈0.78–0.80 nm. The graphene nanochannel consists of rigid walls forming a slit‐like sandwich structure, while the CNT offers cylindrical confinement with a comparable internal diameter. Despite their geometric differences, both systems impose similar spatial restrictions on the fluid, enabling a direct comparison of molecular structuring and transport properties. The extreme confinement enhances wall‐fluid interactions, significantly affecting viscosity and molecular organization. Within the nanochannel, both LJ fluid and water exhibit a single‐file‐like arrangement, with slightly more lateral freedom compared to CNT due to the planar geometry. In contrast, water confined in the CNT forms a highly ordered, chain‐like structure dictated by the cylindrical symmetry and stronger steric constraints. These distinct configurations highlight the strong influence of confinement geometry and dimensionality on fluid behavior at the molecular scale. Detailed analyses of molecular structure, hydrogen bonding, and confinement effects are presented in the following sections.

### 3.1 Structural Organization Under Sub‐Nanometer Confinement

**Figure 2:**
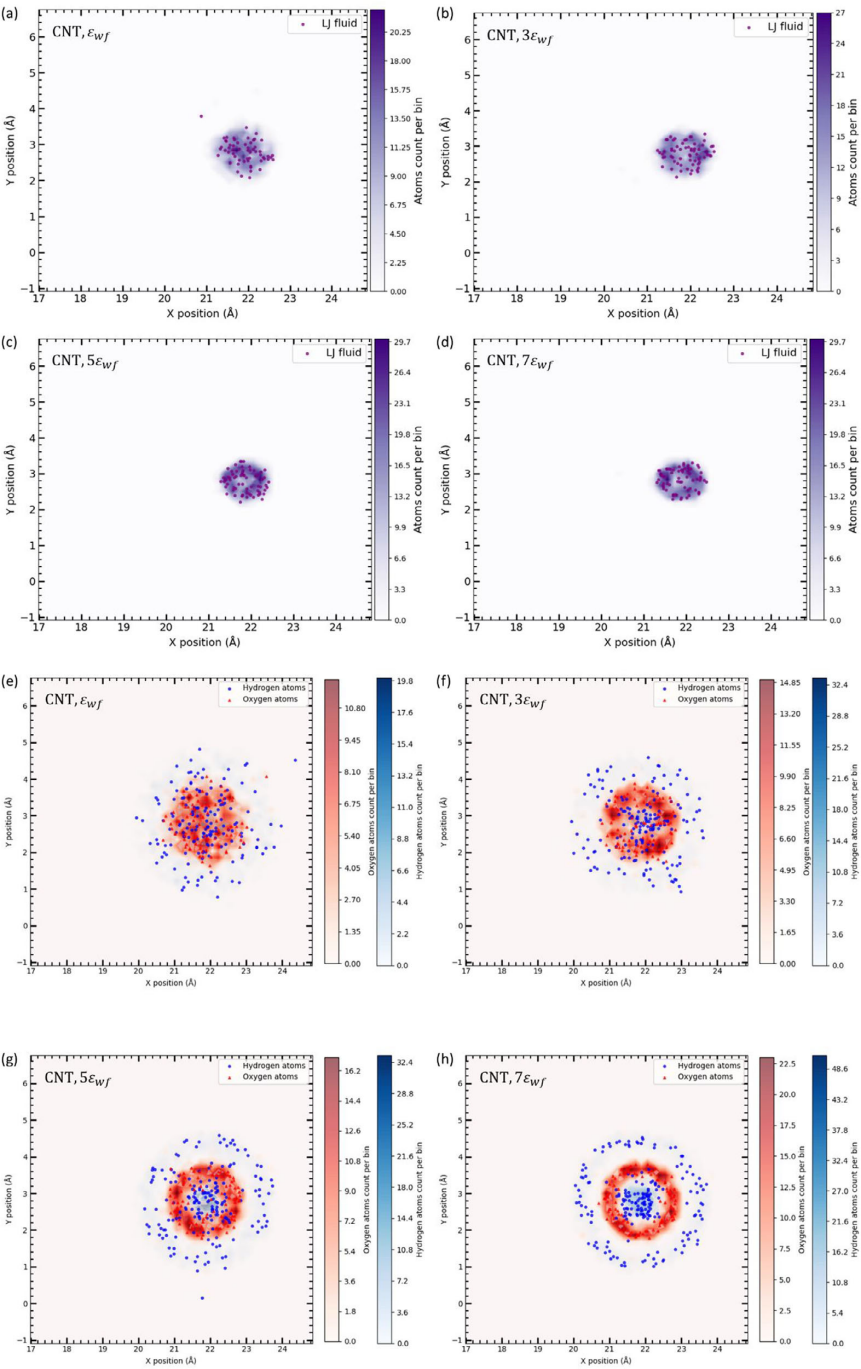
Molecular structuring of confined fluids in carbon nanotubes under varying wall–fluid interactions. a–d) Lennard‐Jones (LJ) fluid evolves from a dispersed to a densely ordered state as wall–fluid interaction strength increases, driven by enhanced van der Waals attraction. e–h) Water molecules exhibit distinct oxygen and hydrogen distributions, forming stable hydrogen‐bonded clusters under strong confinement. In both cases, increased molecular ordering and density near the CNT walls significantly influence nanoscale transport behavior.

**Figure 3:**
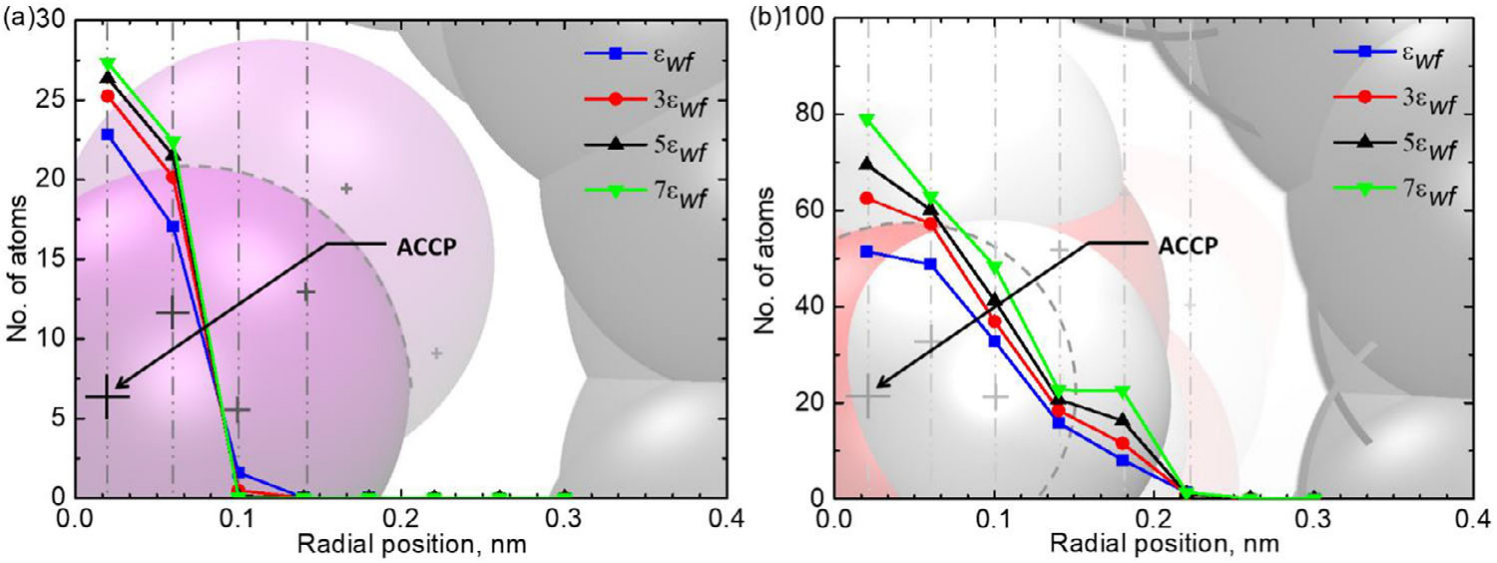
Radial distribution of LJ fluid and water inside the CNT, showing molecular structuring and densification with increasing wall‐fluid interaction strength. a) LJ fluid radial profiles illustrating central localization. b) Water radial profiles highlight increased surface proximity due to polarity effects.

**Figure** **2** presents the molecular distribution of confined fluids within CNTs, highlighting how wall‐fluid interactions influence molecular organization. Figure 2a–d depicts the organization of an LJ fluid inside the CNT as a function of cross‐interaction strength. At lower interaction strengths, the fluid molecules exhibit a more dispersed configuration, indicative of weak adsorption at the CNT surface, allowing enhanced translational mobility. With increasing interaction strength, a pronounced densification of the molecular cluster is observed, suggesting that stronger van der Waals forces promote local structuring within the confinement. This behavior is consistent with prior studies on nanoconfined fluids, which report that strong wall‐fluid interactions induce ordered molecular arrangements, ultimately modulating the effective viscosity and transport properties of confined fluids.^[^ ^61^, ^62^ ^]^ This trend is further quantified in **Figure** **3**, where number of molecules profiles demonstrate enhanced layering near the CNT walls as the interaction strength increases. Such layering, widely observed in confined fluid studies, stems from a balance between cohesive intermolecular forces and adhesive interactions with the solid interface.^[^ ^5^, ^63^ ^]^ The increase in local number of molecules indicates a transition from bulk‐like fluid behavior to a structured interfacial regime, where molecular motion is restricted by spatial constraints. This transition becomes increasingly pronounced in sub‐nm pores, where hydrodynamic properties deviate from classical continuum predictions due to the emergence of discrete molecular interactions. Beyond interaction strength, the nature of wall‐fluid interactions is also sensitive to CNT chirality. Specifically, (8,8) CNTs tend to exert attractive forces on confined molecules, a property that intensifies with increasing chiral index, whereas (6,6) CNTs are known to exhibit repulsive force fields that modulate the flow behavior of encapsulated fluids.^[^ ^64^ ^]^ Beyond these force‐field effects, CNT chirality also governs the structural organization and transport properties of confined water. MD simulations revealed that armchair CNTs, such as the (6,6) type, stabilize highly ordered, single‐file water chains with extended hydrogen‐bond connectivity and large slip lengths, leading to anomalously high flow velocities.^[^ ^62^, ^64^ ^]^ In contrast, zigzag and other chiral CNTs disrupt molecular ordering, enhance interfacial friction, and thereby suppress transport efficiency.^[^ ^5^ ^]^ This chirality‐dependent structuring directly modulates the degree of flow velocity anomaly observed, with armchair CNTs exhibiting the strongest enhancement and other chiralities showing progressively weaker effects. The influence of chirality also diminishes as CNT diameter increases, reflecting a transition toward bulk‐like hydrodynamic behavior in wider tubes. These findings, consistent across atomistic simulations and experiments on CNT membranes,^[^ ^65^ ^]^ highlight that molecular‐scale interactions not only dictate slip length and transport efficiency but also determine the crossover from confinement‐dominated structuring to bulk‐like homogeneous flow as the neutral force region expands in larger pores.

While the LJ fluid highlights general confinement effects, water exhibits additional complexity due to its intrinsic hydrogen bonding capability, as illustrated in Figure 2e–h. Separate visualizations of oxygen and hydrogen atomic distributions reveal that, under higher wall‐fluid interaction strengths, water molecules form stable hydrogen‐bonded clusters. This structuring effect arises from strong hydrogen bonding interactions, which become increasingly prominent under nanoscale confinement. Such interfacial molecular ordering has been widely reported in experimental and computational studies, where confined water layers exhibit significantly altered dielectric and transport properties compared to bulk water.^[^ ^66^, ^67^ ^]^ Enhanced solid‐fluid interactions also increase molecular adsorption at the CNT interface, leading to higher local molecules and deviations from classical hydrodynamic predictions. The spatial heterogeneity of the water cluster distribution reflects the emergence of localized regions of high molecular ordering, a phenomenon with significant implications for confined water transport, ion selectivity, and nanofluidic applications.^[^ ^19^ ^]^

Figure 3 presents the radial distribution of atomic positions for both the LJ fluid and water inside the CNT, providing additional insight into molecular organization under extreme nanoscale confinement. The number of atoms is computed using the Atomic Center Coordinate Point (ACCP) approach, which assigns each atom's position to the central coordinate of its respective radial slab bin. The CNT radius is divided into multiple slabs, with atomic density measured at each bin center to capture the radial molecular distribution. Figure 3a shows the radial atom number profile for the LJ fluid across varying wall‐fluid interaction strengths. The results reveal that atomic population peaks at the CNT center and decreases monotonically toward the walls. This distribution reflects confinement‐induced mobility, where molecules experience minimal wall interactions near the center but increasingly restricted radial motion near the surface due to stronger interfacial forces. Consistent with Figure 2, increasing solid‐fluid interaction strength enhances molecular localization, now evident in the radial distribution. The absence of a bulk‐like region in this sub‐nm system places the entire confined fluid within the interfacial regime, where molecular dynamics are governed by the balance between wall‐fluid forces and self‐organization. As interaction strength increases, central atomic density rises sharply, reflecting enhanced solid‐fluid coupling that drives molecules into low‐energy configurations and structured layers. At sufficiently high interaction strengths, this radial ordering reduces slip lengths and leads to deviations from classical Navier‐Stokes hydrodynamics, as predicted by molecular simulations.^[^ ^5^, ^19^, ^62^, ^64^ ^]^

Figure 3b presents the radial distribution of water molecules inside the CNT, showing a similar overall trend to that of the LJ fluid. As wall‐fluid interactions increase, the number of confined water molecules rises, with the highest density consistently appearing at the central axis of the CNT. However, a key difference emerges: water molecules exhibit a greater tendency to approach the CNT surface compared to the LJ fluid. This behavior is attributed to two fundamental molecular properties. First, the atomic diameter of oxygen is smaller than that of the LJ fluid atoms, allowing water molecules to approach the CNT walls with reduced steric hindrance. Second, and more critically, water's polarity significantly influences its spatial distribution. Unlike the LJ fluid, which consists of neutral atoms, water molecules possess a permanent dipole moment due to their O–H bonds, leading to electrostatic interactions with the CNT surface. Such polar interactions promote partial alignment of water dipoles along the CNT walls, reinforcing the formation of structured interfacial layers. Studies have demonstrated that polar fluids under extreme confinement exhibit orientation‐dependent structuring, where molecules align their dipoles with the confining walls, creating structured interfacial layers.^[^ ^68^ ^]^

### 3.2 Confinement‐Induced Anomalies in Diffusivity and Pseudo‐Viscosity

**Figure 4:**
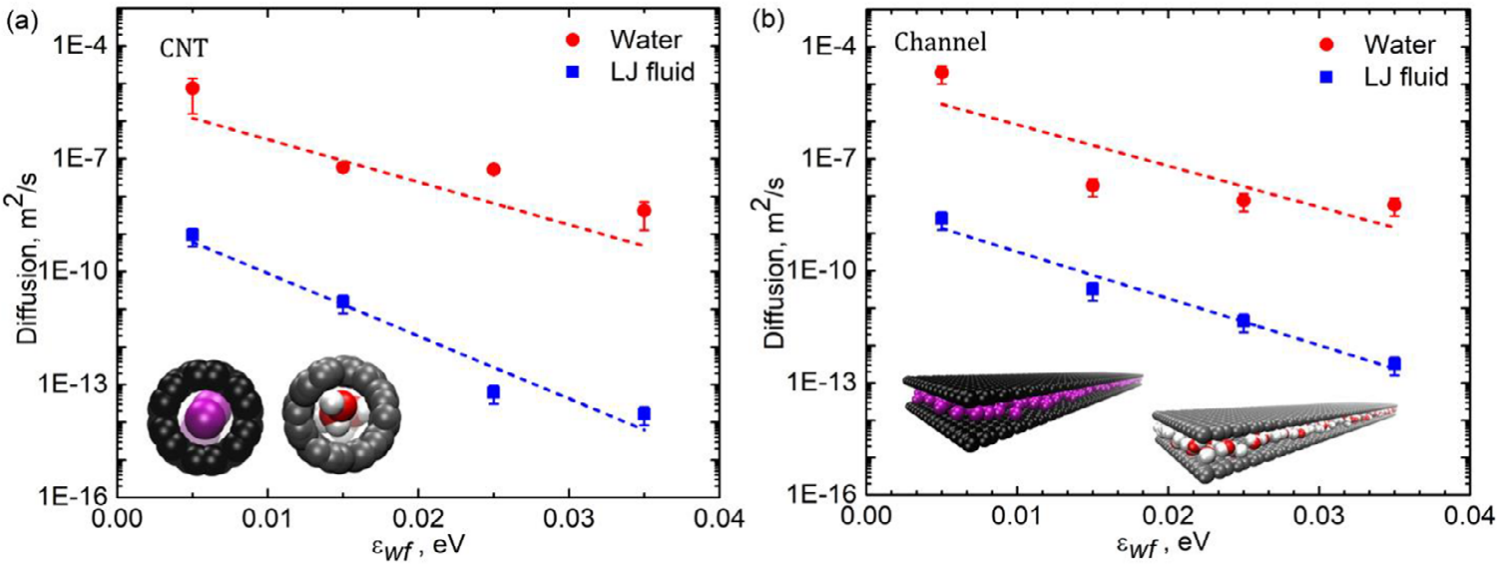
Diffusivity of confined LJ fluid and water as a function of solid‐fluid interaction strength. a) Diffusion trends in CNTs showing confinement‐induced inversion of bulk behavior and enhanced water mobility. b) Diffusion trends in rectangular channels illustrating geometry‐dependent transport and cooperative water diffusion in sub‐nanometer confinement.

**Figure** **4** presents the diffusivity of confined fluids within CNTs and rectangular channels as a function of solid‐fluid interaction strength. Building on the molecular structuring trends observed in Figure 3, this analysis explores how such confinement directly impacts molecular transport. The results indicate a striking deviation from bulk fluid behavior, particularly within sub‐nm confinement, where transport properties are governed by molecular‐scale interactions rather than classical hydrodynamics. Figure 4a illustrates the diffusivity trends within the CNT. Under bulk conditions, LJ fluids typically exhibit higher diffusivity than water due to their weaker intermolecular interactions and the absence of hydrogen bonding networks that hinder molecular mobility. This trend is reflected in diffusion coefficients for bulk fluids, where LJ fluids range from (2.2 to 2.8) × 10^−9^ m^2^ s^−1^, while water modeled by the SPC/E potential falls within (2.3–2.5) × 10^−9^ m^2^ s^−1^. However, under extreme confinement, this expectation is inverted, and water exhibits higher diffusivity than the LJ fluid inside the CNT. This counterintuitive behavior arises from the unique dynamics imposed by molecular ordering, single‐file diffusion, and quantum mechanical effects that emerge at the nanoscale. In sub‐nm CNTs, fluid molecules are constrained to a quasi‐1D arrangement, where steric hindrance governs diffusion. For water, hydrogen bond disruption combined with cooperative, chain‐like motion enhances mobility beyond that of the LJ fluid. In contrast, the LJ fluid, which lacks hydrogen bonding, experiences steric hindrance without benefiting from cooperative motion, leading to reduced diffusivity. Studies have demonstrated that under such confinement, water can exhibit diffusion coefficients exceeding those in bulk conditions, due to the low energy barrier for axial transport along the CNT.^[^ ^13^, ^67^ ^]^ The cylindrical confinement of CNTs imposes additional effects on molecular transport. Due to the curved graphene walls, Pauli repulsion becomes highly significant, particularly in the radial direction, where it limits perpendicular molecular displacement and enforces axial diffusion.^[^ ^69^ ^]^ The quantum mechanical nature of Pauli exclusion interactions has been shown to lower the rotational energy barrier for water molecules, further facilitating their movement along the CNT axis. This effect is particularly significant for polar molecules such as water, as compared to simple nonpolar fluids, which lack electronic polarization effects that could enhance their mobility. Quantum simulations have confirmed that Pauli repulsion in sub‐nm CNTs enhances translational movement along the CNT axis while restricting lateral displacement, reinforcing the observed diffusivity trends.^[^ ^62^ ^]^ As the solid‐fluid interaction strength increases, diffusivity decreases for both fluids due to enhanced fluid structuring at the CNT interface. Strong interactions increase molecular adsorption at the CNT walls, reducing available free volume and increasing frictional resistance. The effect is more pronounced in highly confined CNTs, where surface effects dominate over bulk‐like behavior, aligning with prior studies on nanoscale diffusion suppression due to enhanced interfacial interactions.^[^ ^19^, ^64^ ^]^

Figure 4b illustrates the diffusivity trends within the rectangular channel. Similar to CNTs, water exhibits higher diffusivity than the LJ fluid under sub‐nm confinement, and diffusivity decreases with increasing solid‐fluid interaction strength. However, key differences arise due to the planar nature of the confinement geometry, which affects the diffusion mechanism. Unlike CNTs, where molecular transport follows a highly constrained single‐file motion, rectangular channels provide a broader range of accessible molecular paths, allowing for multi‐file transport and greater lateral mobility. This additional degree of freedom leads to lower sensitivity to Pauli repulsion effects compared to CNTs. For larger pores (>1 nm), diffusion follows a different trend. In this regime, LJ fluids generally exhibit higher diffusivity than water, as weaker intermolecular interactions result in less resistance to movement. Water molecules, in contrast, tend to form structured layers near the channel walls, reducing their effective mobility. However, in sub‐nm confinements, water undergoes a transition to a cooperative single‐file diffusion mechanism, in which molecules move in a highly ordered sequence, minimizing molecular resistance and enhancing axial diffusion. This cooperative nature of water diffusion enables it to surpass LJ fluid diffusivity, which becomes increasingly hindered by steric effects under extreme confinement.^[^ ^70^, ^71^ ^]^ In both CNTs and rectangular channels, confinement effects fundamentally alter diffusion behavior, with key distinctions observed based on pore geometry, fluid‐wall interactions, and molecular structure. Overall, the enhanced water diffusivity observed under extreme confinement highlights the governing influence of hydrogen bonding, cooperative transport mechanisms, and quantum effects on nanoscale fluid dynamics.

**Figure 5:**
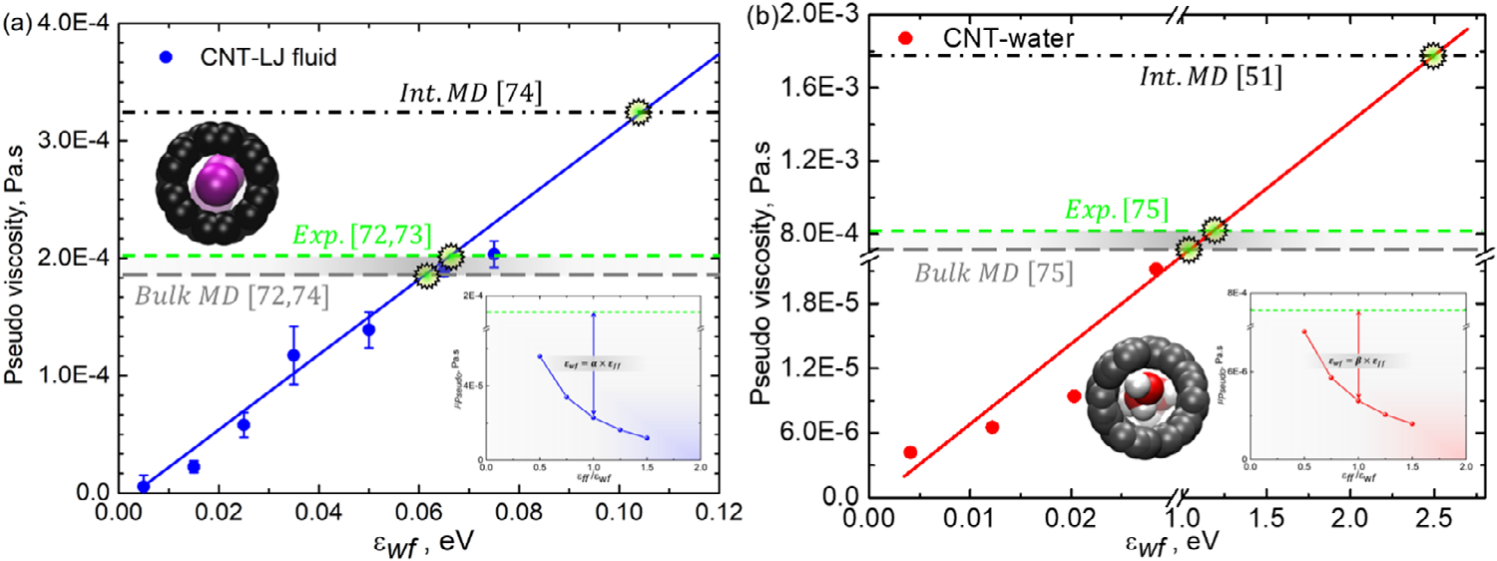
Pseudo‐viscosity trends of confined LJ fluid and water inside sub‐nanometer CNTs as a function of wall‐fluid interaction strength. a) LJ fluid shows reduced pseudo‐viscosity compared to bulk due to slip flow and molecular structuring. b) Water exhibits lower pseudo‐viscosity driven by polarity, hydrogen bonding, and cooperative transport under extreme confinement.

**Figure** **5** presents the viscosity of both an LJ fluid and water confined within sub‐nm CNTs. However, due to the extreme confinement at this scale, viscosity cannot be defined in the conventional macroscopic sense. Instead, we refer to it as pseudo‐viscosity, acknowledging that fluid properties are significantly altered in the sub‐nm regime. The concept of pseudo‐viscosity serves as an effective metric for describing resistance to flow under extreme confinement, where conventional fluid behavior governed by continuum mechanics is no longer valid. While we analyze pseudo‐viscosity using the Hagen–Poiseuille equation as a reference, it remains a hypothetical construct offering insights into molecular transport dynamics at the nanoscale. Figure 5a illustrates the pseudo‐viscosity for the LJ fluid inside the CNT. The results indicate that as wall‐fluid interaction strength increases, pseudo‐viscosity also increases, which aligns with expectations. However, a notable observation is that pseudo‐viscosity inside the CNT remains significantly lower than its bulk counterpart. We compare our results with experimental data,^[^ ^72^, ^73^ ^]^ bulk‐phase values,^[^ ^72^, ^74^ ^]^ and interfacial^[^ ^74^ ^]^ viscosities reported in molecular dynamics simulations. Typically, near solid surfaces, viscosity increases due to stronger wall‐fluid interactions, but here, the opposite trend is observed. Previous studies have shown that in extreme confinement, molecular mobility is enhanced by slip‐flow effects, leading to viscosity values lower than bulk predictions.^[^ ^19^, ^62^ ^]^ Further analysis reveals that when the wall‐fluid interaction parameter is ≈13 to 15 times greater than the fluid‐fluid interaction, pseudo‐viscosity approaches the bulk viscosity of the LJ fluid. This suggests that in sub‐nm confinements, fluid‐wall interactions and structural constraints dominate transport resistance, reducing surface‐induced drag and accelerating fluid movement. Additionally, when interaction strength approaches 21 times the fluid‐fluid interaction, pseudo‐viscosity aligns with interfacial viscosity values reported in bulk‐like systems from MD simulations. These findings highlight the importance of molecular structuring, interfacial forces, and nanoscale slip flow in determining viscosity under extreme confinement.^[^ ^61^ ^]^ To further investigate this behavior, we analyze pseudo‐viscosity based on the ratio of fluid‐fluid to fluid‐wall interactions. Initially, it is hypothesized that a ratio of one would yield bulk‐like viscosity due to isotropic interactions. However, the analysis reveals significant deviation from this expectation. This deviation results from geometric constraints imposed by the CNT, which alter local molecular arrangements and molecular alignment, impacting viscosity even when interaction energies resemble bulk conditions. The results indicate that when the wall‐fluid interaction strength is α times greater than the fluid‐fluid interaction, pseudo‐viscosity aligns with bulk experimental values—reinforcing the role of geometric confinement and molecular structuring in governing nanoscale fluid transport properties.

Figure 5b presents the pseudo‐viscosity for water inside the sub‐nm CNT. Similar to the LJ fluid, pseudo‐viscosity increases with increasing wall‐fluid interaction strength. However, due to water's polarity and strong hydrogen bonding, its pseudo‐viscosity remains markedly lower than both its bulk, experimental value^[^ ^75^ ^]^ and interfacial viscosity reported in larger nanoconfined systems.^[^ ^51^ ^]^ This trend is consistent with previous findings that confined water exhibits ultralow friction and enhanced molecular transport in CNTs, facilitated by cooperative hydrogen bonding and structural ordering.^[^ ^13^, ^67^ ^]^

To contextualize these computational findings, although this work does not present original experimental data, the pseudo‐continuum behavior and pseudo‐viscosity elucidated through MD simulations are strongly supported by existing nanoscale fluid transport experiments. Prior studies in nanofluidics and CNT flows have demonstrated enhanced slip lengths, significantly reduced interfacial friction, and measurable deviations from bulk viscosity, all consistent with the theoretical framework developed.^[^ ^67^, ^76^ ^]^ These empirical observations provide compelling indirect validation of the molecular mechanisms we describe. Experimental validation through techniques such as nanofluidic rheometry, nanoscale velocimetry, or atomic force microscopy–based friction measurements could further quantify slip behavior and effective viscosity in confined geometries, thereby directly substantiating the pseudo‐continuum model proposed. While direct experimental verification remains outside the scope of this computational study, these advanced methodologies offer a clear pathway for future work to bridge simulation and experiment.

### 3.3 Sub‐Nanometer Flow Patterns in Confined Fluids

**Figure 6:**
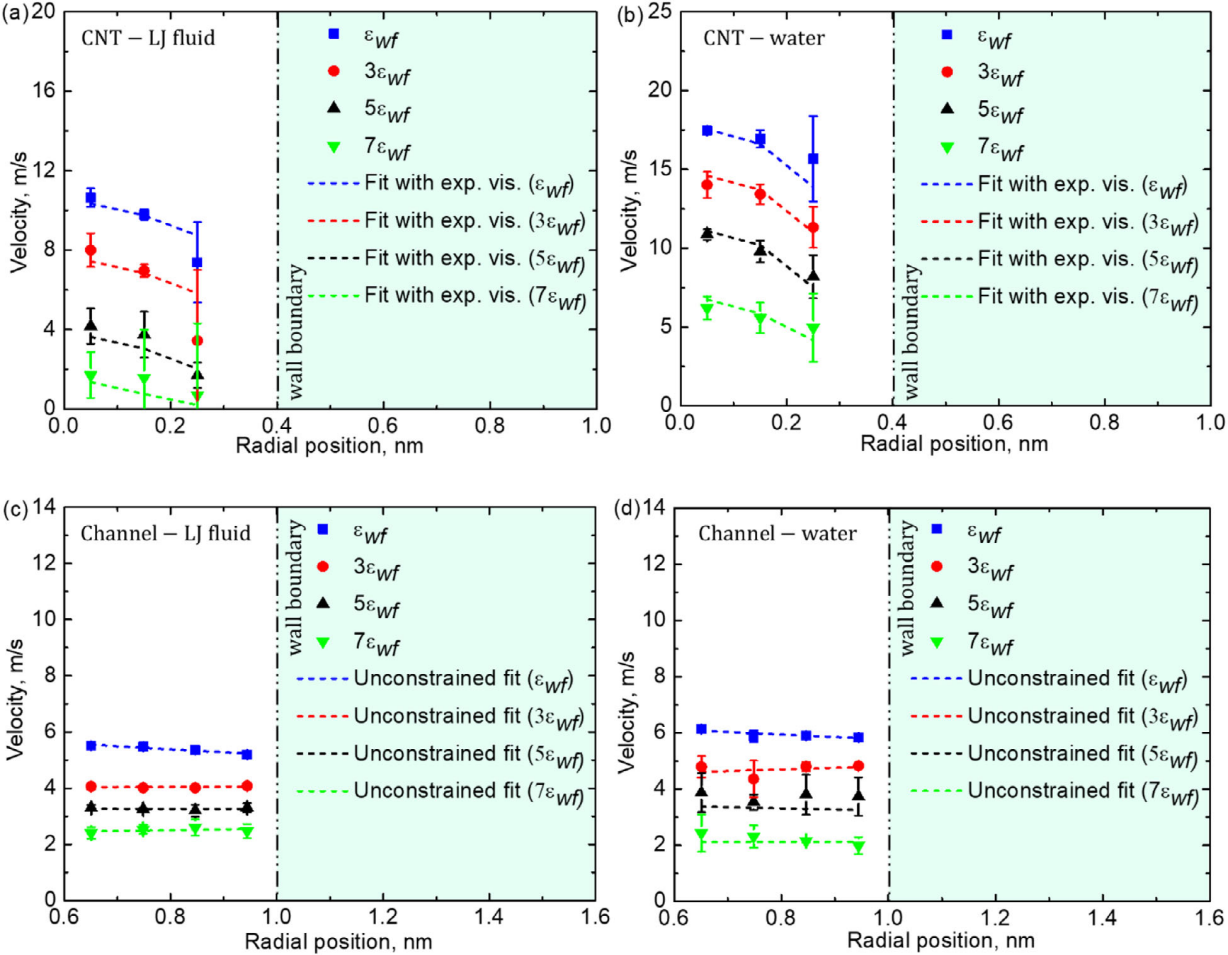
Velocity profiles of LJ fluid and water in sub‐nanometer CNTs and rectangular channels under varying wall‐fluid interaction strengths: a) LJ fluid in a CNT, b) water in a CNT, c) LJ fluid in a rectangular channel, and d) water in a rectangular channel.

**Figure** **6** illustrates the velocity profiles of confined fluids within sub‐nm geometries, capturing the influence of molecular‐scale fluctuations and wall‐fluid interactions on flow behavior. Building on the pseudo‐viscosity trends observed in Figure 5, this analysis explores how extreme confinement alters velocity distributions beyond classical hydrodynamic predictions. Figure 6a presents the velocity profile of an LJ fluid confined within a sub‐nm CNT. Under such confinement, molecular‐scale fluctuations dominate the velocity distribution, making it difficult to classify the profile as distinctly parabolic or plug‐like. However, within circular geometries, the overall trend approximates a parabolic shape, consistent with prior MD studies of nanoconfined fluids.^[^ ^77^ ^]^ Increasing wall‐fluid interaction strength enhances velocity fluctuations and reduces overall flow velocity, consistent with nanoconfined transport observations.^[^ ^5^ ^]^ This reduction arises from dominant van der Waals forces at the interface, imposing resistance to molecular motion. Notably, as interaction strength increases, the velocity profile transitions toward a more uniform distribution, suggesting that interfacial interactions stabilize atomic arrangements and reduce velocity deviations. Enhanced molecular binding near the walls leads to a more stabilized center‐of‐mass motion, aligning with experimental studies of confined transport.^[^ ^67^ ^]^ Despite nanoscale confinement limiting continuum assumptions, MD results are fitted to experimental viscosity data using a parabolic fitting approach, as the general trend aligns most closely with a parabolic profile. However, the confinement scale imposes limitations on classical continuum assumptions, as only single‐file molecular transport occurs in this regime.^[^ ^78^ ^]^ The discrete molecular nature of flow produces complex velocity distributions with dynamic atomic fluctuations in all spatial directions. Ensemble averaging reveals a parabolic tendency, although with local deviations due to atomic‐scale effects.

Figure 6b shows the velocity profile of confined water, exhibiting similar fluctuations but aligning more closely with a parabolic shape than the LJ fluid. This behavior is attributed to water's polarity and hydrogen bonding, which introduce additional intermolecular structuring. These effects influence the velocity profile, producing flow patterns resembling continuum‐scale predictions.^[^ ^79^ ^]^ Even in sub‐nm confinement, water molecules exhibit cooperative transport behaviors that enhance the stability of the velocity profile, leading to a structured molecular arrangement consistent with hydrodynamic theory at larger scales. Atomic center coordinate analysis reveals dynamic molecular motion with frequent positional rearrangements. Although increasing wall‐fluid interaction strength reduces velocity in both fluids, water maintains higher flow velocity due to its structured hydrogen‐bonding network, which minimizes momentum loss at the interface — a distinct behavior not observed in macroscopic fluids.^[^ ^10^ ^]^ As the interaction strength increases, the velocity profile transitions further toward a flatter shape, mirroring the behavior observed in the LJ fluid case. Despite the intrinsic fluctuations, the parabolic tendency remains a reasonable approximation. However, at sufficiently high interaction strengths, the velocity profile appears nearly linear, suggesting a transition from parabolic to plug‐like behavior. This is consistent with theoretical predictions for molecular flow under extreme spatial constraints, where interactions at the solid‐fluid interface suppress shear‐driven velocity gradients.^[^ ^80^ ^]^ Pseudo‐continuum behavior occurs primarily under extreme nanoconfinement where channel sizes approach sub‐nanometer dimensions, typically near or below 1 nm, allowing molecular discreteness to influence flow while ensemble‐averaged velocity profiles still resemble classical continuum forms. Our simulations show that pseudo‐continuum dynamics depend strongly on fluid type, with polar fluids such as water exhibiting distinct behavior due to hydrogen bonding and strong wall‐fluid interactions, whereas nonpolar Lennard‐Jones fluids show similar trends governed by van der Waals forces and steric effects. Channel geometry, particularly curvature, as in CNT versus planar graphene channels, further modulates pseudo‐continuum characteristics by affecting molecular ordering and slip length. While this study establishes pseudo‐continuum flow under several specific conditions, a comprehensive exploration of critical parameters including channel size, fluid polarity, and surface chemistry remains an important target for future research. Experimentally, direct observation of pseudo‐continuum dynamics is challenging but supported indirectly by measurements of enhanced slip and anomalous viscosity in nanoconfined fluids. Emerging techniques such as nanoscale velocimetry, atomic force microscopy‐based friction assays, and nanorheological measurements offer promising avenues to more directly verify and characterize pseudo‐continuum behavior, bridging simulation predictions and experimental validation to advance nanofluidic science.

Complementing these experimental efforts, our current molecular dynamics simulations demonstrate pseudo‐continuum velocity profiles under selected pressure gradients and fluid densities representative of nanoconfined flows. While systematic mapping of the critical conditions for the emergence of this behavior by varying pressure and density remains a future objective, such simulations are feasible and anticipated to elucidate how these parameters influence molecular layering, slip, and velocity distributions. Preliminary results indicate that increased pressure gradients and fluid density variations significantly affect nanoscale transport features. Ongoing studies aim to quantitatively identify critical thresholds and underlying mechanisms, ultimately providing a framework to guide and interpret targeted experimental investigations.

In Figure 6c, the velocity profile for a LJ fluid in a sub‐nm rectangular channel is presented, with Figure 6d illustrating the corresponding behavior for polarized water. A systematic analysis of interaction strength variations reveals that increasing wall‐fluid interactions leads to a reduction in flow velocity in both cases, consistent with the behavior observed in CNTs. However, the velocity distribution in rectangular confinement exhibits notable deviations from the circular geometry case. The highly confined nature of the rectangular geometry results in enhanced fluctuations, making it challenging to precisely classify the velocity profile as parabolic or plug‐type. One particularly intriguing finding is that in rectangular geometries, the velocity distribution appears significantly closer to a flat profile compared to CNTs. This observation is further supported by unconstrained fitting methods, which reveal that increasing interaction strength causes a transition toward a nearly linear velocity distribution. The physical origin of this effect is attributed to differential surface interactions. In a circular geometry, molecular transport is subject to uniform surface forces in all directions, promoting a parabolic flow tendency. Conversely, in rectangular confinement, atoms experience asymmetric interactions due to surface boundaries on two opposing sides, leading to an overall flattening of the velocity distribution. This effect has been previously reported in studies of fluid transport in nano‐slits and graphene nanocapillaries, where molecular layering effects induce distinct deviations from classical hydrodynamics.^[^ ^70^ ^]^ The rectangular geometry exhibits reduced molecular surface contamination compared to CNTs, leading to differences in velocity structuring. Unlike the cylindrical case, where the fluid is subject to uniform confinement forces, the asymmetric boundaries of rectangular channels modify the velocity profile, allowing it to resemble bulk‐like transport behavior at larger scales. This distinction has important implications for nanofluidic applications, as it suggests that molecular transport properties can be significantly modulated by confinement geometry, in agreement with prior experimental observations on water transport through 2D nano‐conduits.^[^ ^81^ ^]^

### 3.4 Hydrogen‐Bond Disruption and Molecular Insights in Ultra‐Confined Environments

**Figure 7:**
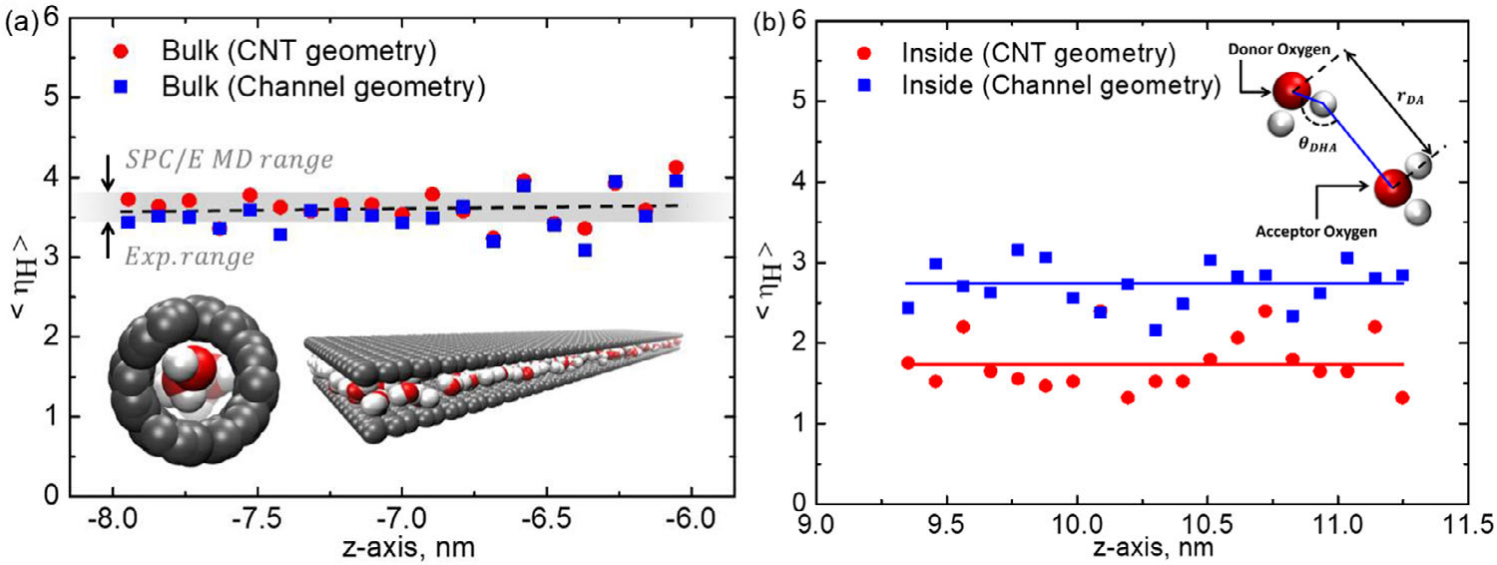
Hydrogen bond analysis of confined water in sub‐nanometer geometries. a) H‐bond distribution in the reservoir region and b) H‐bond distribution inside CNT and rectangular channels, demonstrating the influence of confinement on hydrogen bond networks.

**Figure** **7** presents the hydrogen bond (H‐bond) analysis for polarized water within nanoconfinement, comparing both the reservoir and confined regions for the rectangular and CNT geometries. In Figure 7a, the H‐bond distribution at the reservoir is analyzed for both geometries. The results indicate that within the bulk‐like region of the reservoir, the H‐bond formation remains nearly identical in both cases, confirming that geometric confinement has no significant influence on hydrogen bonding outside the confinement region. The spatial distribution of H‐bonds at the reservoir follows a consistent trend, which aligns well with both experimental and MD simulations for the SPC/E water model.^[^ ^53^, ^82^, ^83^ ^]^ While minor fluctuations in the H‐bond count are observed with distance, the overall average remains within the expected range reported in prior experimental and computational studies.^[^ ^82^ ^]^

Following the validation of H‐bond behavior in the bulk‐like reservoir region, the focus shifts to the confined water molecules inside the CNT and rectangular geometries, as depicted in Figure 7b. Unlike in the reservoir, a notable difference in H‐bond distribution emerges between the two geometries, demonstrating the impact of nanoconfinement on molecular interactions. The overall number of H‐bonds within confinement is significantly reduced compared to the bulk phase, consistent with previous findings on water structure under extreme spatial constraints.^[^ ^84^ ^]^ This reduction in hydrogen bonding results from the steric limitations imposed by the confining walls, which restrict the orientational flexibility required for optimal H‐bond formation. We found that the H‐bond density is higher within the rectangular geometry compared to the CNT. This difference arises due to the distinct molecular arrangement dictated by the confinement. In the rectangular geometry, water molecules predominantly adopt a layered or quasi‐single‐file arrangement, with two parallel interfaces interacting with the graphene walls. This structured configuration promotes relatively higher hydrogen bonding compared to the CNT case, where water molecules follow a more constrained single‐chain‐like arrangement due to the cylindrical symmetry of the nanotube. Prior studies have demonstrated that such geometric constraints significantly alter the hydrogen bond network, leading to anisotropic structuring effects that differ from bulk‐phase behavior.^[^ ^85^ ^]^ These findings provide further evidence that at extreme confinement scales, H‐bond breakdown occurs, which directly influences the fluid's transport properties. The disruption of the hydrogen bond network plays an important role in determining the viscosity, flow dynamics, and molecular mobility within the confined space. This impact on flow behavior will be further explored in subsequent analyses. Although our present study captures hydrogen bond disruption under fixed temperature, pressure, and chemically uniform interface conditions, it is important to note that these factors critically influence hydrogen bonding dynamics and interfacial interactions. Variations in temperature can strengthen or weaken hydrogen bonds by altering molecular motion, thus potentially modifying flow rate differences between water and Lennard‐Jones fluids. Similarly, pressure changes affect fluid density and molecular packing, impacting hydrogen bonding and interfacial repulsion. Additionally, chemical modifications of the confining interface, such as changes in surface functionalization or charge, can significantly alter interfacial Pauli exclusion effects and slip behavior. Quantitative separation and assessment of the relative contributions of hydrogen bond disruption and Pauli repulsion require systematic simulations varying temperature, pressure, and interface chemistry. Such studies are essential to fully elucidate their respective roles in nanoconfined fluid transport.

**Figure 8:**
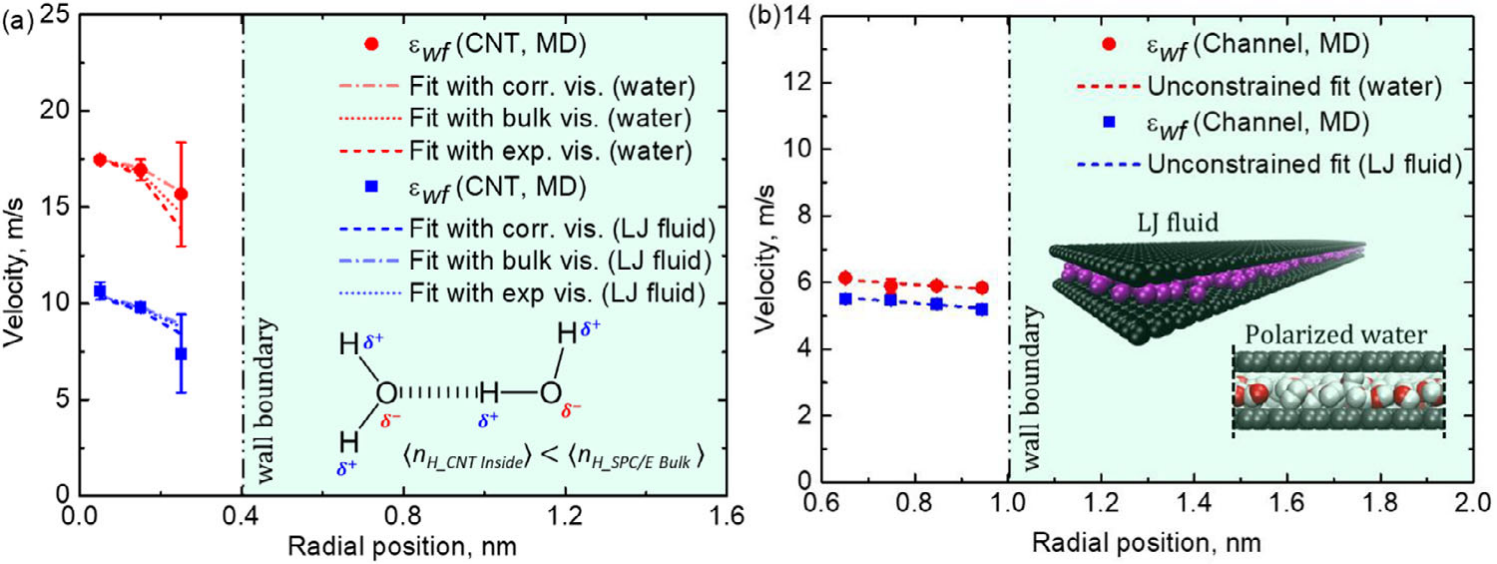
Velocity profiles of LJ fluid and water in a) CNTs and b) rectangular channels as a function of wall‐fluid interaction strength.

**Figure** **8** presents a comparative analysis of van der Waals and Coulombic interactions under sub‐nanometer confinement, highlighting their dominant role in governing fluid transport. Results are shown for CNT confinement (a) and rectangular nanochannel confinement (b). The velocity profiles of the LJ fluid and polarized water are compared, revealing that water flows approximately two to three times faster than the LJ fluid under identical conditions, consistent with experiments and simulations reporting 2 − 4 × faster water transport through sub‐nm confinements.^[^ ^13^, ^76^, ^86^ ^]^ This effect is more pronounced in CNTs than in rectangular geometries, indicating that curvature enhances slip and transport. Previous studies have attributed the ultra‐fast flow of water to reduced interfacial friction (large slip lengths) resulting from disrupted hydrogen bonding and hydrophobic, atomically smooth graphene surfaces.^[^ ^10^, ^87^ ^]^ Our findings extend this explanation while extending it by identifying additional mechanisms, including confinement‐induced viscosity reduction and strong Pauli exclusion repulsion at the fluid–solid interface, which together accelerate molecular motion beyond classical hydrodynamic predictions.^[^ ^78^, ^88^ ^]^ A fundamental distinction between LJ fluids and water emerges in this regime: while nonpolar fluids often exhibit higher bulk mobility due to weaker cohesive interactions, under sub‐nanometer confinement, water becomes faster because hydrogen‐bond disruption reduces intermolecular constraints, dramatically enhancing translational freedom. Water's polarity further intensifies Pauli repulsion with channel walls, lowering interfacial resistance relative to LJ fluids,^[^ ^13^, ^78^ ^]^ providing a mechanistic basis for why confined water can surpass LJ fluids in flow rate, an effect validated by advanced simulations benchmarked against ab initio results.^[^ ^89^ ^]^ Our molecular dynamics analysis indicates that hydrogen‐bond disruption is the dominant factor facilitating this anomalous mobility, while interface‐mediated Pauli exclusion acts as a secondary but important amplifier by lowering wall friction. Although their quantitative contributions cannot yet be isolated, the strong correlation observed between reduced hydrogen‐bond counts and increased velocity supports this interpretation. Systematic studies involving temperature variation to tune hydrogen‐bond stability and interfacial modifications to regulate repulsion strength will be essential to fully disentangle their roles.^[^ ^90^, ^91^ ^]^ Additionally, the low molecular mass of water compared to the LJ fluid enhances its dynamic response under confinement, further contributing to the observed velocity differences. Altogether, these results demonstrate that classical transport theories break down at the molecular scale, where transport is governed by a synergistic interplay of hydrogen‐bond collapse, interfacial quantum repulsion, molecular polarity, and mass effects.

The influence of confinement geometry is examined by comparing CNTs and rectangular nanochannels, revealing systematically lower velocities in rectangular channels than in CNTs as a direct consequence of curvature effects.^[^ ^38^ ^]^ In CNTs, continuous curvature enhances Pauli repulsion, facilitates favorable molecular ordering, and minimizes steric hindrances.^[^ ^13^ ^]^ Conversely, rectangular geometries impose greater molecular structuring constraints, reducing the impact of repulsive forces and yielding lower flow velocities. Although our simulations focus on sub‐nanometer channels near 1 nm, minor variations in pore diameter at or below this scale can significantly influence curvature‐dependent fluid transport. Smaller CNT diameters amplify curvature effects, promoting stronger molecular ordering, longer slip lengths, and enhanced interfacial repulsion, which collectively accelerate flow. As the channel size approaches or falls below 1 nm, confinement‐induced structuring becomes more discrete and size‐sensitive, potentially modifying slip behavior and transport efficiency more sharply than in larger pores. Key geometric parameters such as radius of curvature and effective confinement cross‐section critically govern how curvature modulates molecular arrangement and dynamic properties. Thus, small size variations induce nuanced shifts in hydrogen‐bond network disruption and Pauli repulsion effects, impacting flow rates. While our current results illustrate general curvature trends, systematic investigations spanning sub‐1‐nm channel sizes would be invaluable for quantifying fluid transport sensitivity to nanoscale geometry and fully elucidating curvature's role across different confinement regimes.

Importantly, despite highly discrete molecular structuring within the confined fluid, velocity profiles approaching continuum parabolic shapes emerge—a phenomenon known as pseudo‐continuum behavior. This denotes a regime under sub‐nanometer confinement where fluid motion, though governed by discrete molecular interactions and strong statistical fluctuations, exhibits ensemble‐averaged velocity profiles resembling the smooth parabolic distributions predicted by continuum hydrodynamics. This apparent macroscopic resemblance arises from averaging molecular‐scale fluctuations, producing an illusion of continuum flow despite the known breakdown of bulk hydrodynamic assumptions. Such pseudo‐continuum behavior is consistent with molecular dynamics and nanofluidic flow experiments demonstrating continuum‐like velocity fields even at sub‐nanometer length scales.^[^ ^78^, ^92^ ^]^ These observations, combined with the curvature‐related findings above, highlight the critical role of molecular ordering in confined transport and provide new insights into the mechanisms governing nanoconfined fluid flow. Our results challenge conventional assumptions about viscosity, flow behavior, and interfacial forces at molecular scales, underscoring the need for a paradigm shift in modeling transport phenomena in sub‐nanometer geometries with implications for nanofluidics, selective separations, and energy‐efficient water transport technologies.

## 4 Conclusion

In summary, molecular dynamics simulations elucidate the molecular transport mechanisms of Lennard‐Jones fluids and polar water confined within sub‐nanometer carbonaceous nanoconfinements, including cylindrical carbon nanotubes and graphene‐based rectangular nanochannels. Our results reveal that extreme spatial confinement induces pronounced deviations from classical hydrodynamic predictions, driven by viscosity reduction, hydrogen‐bond network disruption, and intensified interfacial molecular interactions. Distinct transport regimes emerge: nonpolar Lennard‐Jones fluids exhibit velocity attenuation with increasing wall‐fluid interactions, whereas polar water molecules sustain enhanced flow velocities, inverting bulk‐phase trends. This anomalous enhancement arises from hydrogen‐bond network breakdown, which diminishes intermolecular resistance and augments molecular mobility, further amplified by Pauli exclusion effects at the fluid‐solid interface. Curvature effects in carbon nanotubes intensify these mechanisms, facilitating accelerated transport compared to planar graphene channels, where geometric constraints induce enhanced molecular ordering and attenuate flow efficiency. Hydrogen‐bonding analysis corroborates these observations, revealing more significant network disruption in cylindrical confinement, which directly modulates viscosity and flow dynamics, providing a molecular‐level rationale for the superior water transport in nanotubes. Additionally, water's lower molecular mass enhances its dynamic response under nanoconfinement relative to Lennard‐Jones fluids. Collectively, fluid transport at the sub‐nanometer scale is governed by the synergistic interplay of hydrogen‐bond dynamics, interfacial forces, curvature‐induced molecular ordering, and molecular mass. These mechanisms dominate nanoscale transport, rendering continuum models inadequate for predicting flow in atomic‐scale confinements. Notably, certain regimes exhibit pseudo‐continuum behavior, where velocity profiles and transport trends retain mesoscopic characteristics despite molecular‐scale effects. These findings establish a theoretical framework for extending continuum fluid concepts to the atomistic regime, providing mechanistic insights essential for advancing predictive models of nanoscale transport. This framework guides the rational design of next‐generation nanofluidic and separation technologies with broad applications in ultra‐efficient water purification, selective ion sieving, nanofluidic energy harvesting, and biomolecular sensing. By understanding and harnessing molecular‐scale transport phenomena, engineering of nanostructured materials with unprecedented transport efficiencies and selectivity becomes possible, promising transformative impacts in environmental sustainability, clean energy, and precision medicine.

## Conflict of Interest

The authors declare no conflict of interest.

## Data Availability

The data that support the findings of this study are available from the corresponding author upon reasonable request.
